# Assessing the effect of acid and alkali treatment on a halloysite-based catalyst for dry reforming of methane†

**DOI:** 10.1039/d3ra07990b

**Published:** 2024-02-05

**Authors:** Ahmed Abotaleb, Dema Al-Masri, Alaa Alkhateb, Kamal Mroue, Atef Zekri, Yasmin Mashhour, Alessandro Sinopoli

**Affiliations:** a Qatar Environment and Energy Research Institute, Hamad Bin Khalifa University P.O. Box 34110 Doha Qatar asinopoli@hbku.edu.qa; b Earthna Center for a Sustainable Future, Qatar Foundation Doha Qatar; c HBKU Core Labs, Hamad Bin Khalifa University P.O. Box 34110 Doha Qatar; d Department of Chemistry and Earth Sciences, College of Arts and Sciences, Qatar University Doha P.O. Box 2713 Qatar

## Abstract

Dry reforming of methane (DRM) has recently received wide attention owing to its outstanding performance in the reduction and conversion of CH_4_ and CO_2_ to syngas (H_2_ and CO). From an industrial perspective, nickel (Ni)-supported catalysts have been deemed among the most suitable catalysts for DRM owing to their low cost and high activity compared to noble metals. However, a downside of nickel catalysts is their high susceptibility to deactivation due to coke formation and sintering at high temperatures. Using appropriate supports and preparation methods plays a major role in improving the activity and stability of Ni-supported catalysts. Halloysite nanotubes (HNTs) are largely utilized in catalysis as a support for Ni owing to their abundance, low cost, and ease of preparation. The treatment of HNTs (chemical or physical) prior to doping with Ni is considered a suitable method for increasing the overall performance of the catalyst. In this study, the surface of HNTs was activated with acids (HNO_3_ and H_2_SO_4_) and alkalis (NaOH and Na_2_CO_3_ + NaNO_3_) prior to Ni doping to assess the effects of support treatment on the stability, activity, and longevity of the catalyst. Nickel catalysts on raw HNT, acid-treated HNT, and alkali-treated HNT supports were prepared *via* wet impregnation. A detailed characterization of the catalysts was conducted using X-ray diffraction (XRD), BET surface area analysis, scanning electron microscopy (SEM), transmission electron microscopy (TEM), solid-state nuclear magnetic resonance (ssNMR), H_2_-temperature programmed reduction, (H_2_-TPR), CO_2_-temperature programmed desorption (CO_2_-TPD), and Ni-dispersion *via* H_2_-pulse chemisorption. Our results reveal a clear alteration in the structure of HNTs after treatment, while elemental mapping shows a uniform distribution of Ni throughout all the different supports. Moreover, the supports treated with a molten salt method resulted in the overall highest CO_2_ and CH_4_ conversion among the studied catalysts and exhibited high stability over 24 hours testing.

## Abstract 2

Aluminosilicate nanotubular clays (HNTs) for methane dry reforming have recently received wide attention. Acid-treated HNTs show enhanced surface area and active sites, while alkali-treated ones exhibit improved stability and CO_2_ adsorption.

## Graphical abstract

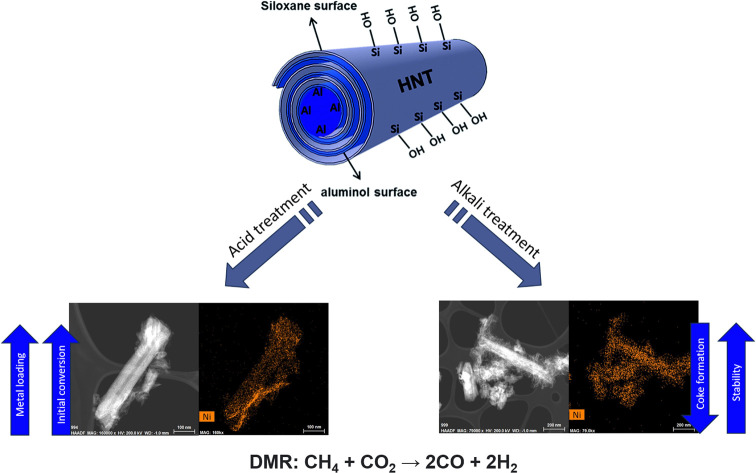

## 1. Introduction

Carbon dioxide (CO_2_) and methane (CH_4_) have been identified by the scientific community as two of the main greenhouse gases responsible for the global climate change. With the global average temperature increasing and the frequent occurrence of environmental disasters, it has become urgent to mitigate greenhouse gases and greenhouse gas effects on a global scale. In this regard, dry reforming of methane (DRM) represents a valuable process for the transformation of methane and carbon dioxide into valuable synthetic gas (syngas) comprising H_2_ and CO, with an H_2_ : CO molar ratio close to unity. The produced syngas can be used as a feedstock in the synthesis of higher hydrocarbons in the Fischer–Tropsch process.^1–4^ The DRM reaction requires copious amounts of energy, with reaction temperatures in the range 550–900 °C. However, side reactions such as methane decomposition, Boudouard, and reverse water–gas shift (RWGS) reactions, are responsible for coke deposition (hence catalyst deactivation) and low product selectivity. To overcome these side reactions, noble metals such as Rh, Ru, Pt and Pd have been largely utilized as catalysts for DRM,^5–8^ although the rarity and high cost of noble metals impose some limitations on their adoption at a large scale.^9–11^ Consequently, over the last decade, nickel has attracted significant attention in the catalysis community as an ideal candidate for replacing noble metals in the industry owing to its high activity, good C–H bond breaking ability, and cost-effectiveness.^12–16^ Although nickel may solve some cost and material availability issues, Ni-based catalysts are prone to coke formation and sintering at high temperatures.^17^

Various strategies have been investigated to enhance the stability of nickel catalysts by tuning their morphology and electronic properties.^5,18^ For example, using nickel in the presence of other co-catalysts/promoters (typically transition metals) has been investigated by Li *et al.* by doping silica substrate with La^3+^ ions,^19–22^ resulting in improved activity and stability of Ni-based catalysts. Similarly, Alotaibi *et al.* evaluated the effect of La and Ca as promoters for Ni-based zeolite catalysts for DRM, demonstrating a higher stability for the La-containing catalyst.^23^ Developing core–shell structured Ni-based catalysts is another strategy for improving catalytic performance, attributed to the confinement effect and the high dispersion of the catalyst. In fact, the presence of an outer layer shell can prevent coke deposition on top of the active metal phase.^20,24^ An elegant example that succeeded in confining the active nickel into the channels of mesoporous silica has been reported by Xie *et al.*^25–31^ Core–shell structure catalysts have been gradually developed, and nickel has been encapsulated, or confined, in well-defined structures, such as perovskites,^32,33^ pyrochlores,^34^ fluorites,^35^ hexaaluminates,^36^ spinels,^37^ and sandwich-like structures.^38,39^ Additionally, the size of a nickel particles was found to strongly influence the coke formation, as reported by Gould *et al.*, who demonstrated that 5 nm is the ideal size to enhance the coking-resistance performance of a nickel catalyst.^40–43^ Despite the large number of approaches and investigations, coke formation and sintering still hinder the adoption of Ni-based catalysts in practical and industrial applications.^20,24^

The development of novel catalytic supports has always become more relevant within the context of sustainable development and cyclic economy. In this regard, natural clays have recently been exploited in various catalytic applications owing to their abundancy, high thermal conductivity, good surface area, and low cost.^44–51^ Among the natural clays, halloysites (HNT), characterized by the molecular formula Al_2_SiO_2_(OH)_4_·*n*H_2_O and a two-layer structure, have been extensively studied. The outer layer of HNTs comprises a tetrahedral silicon-oxygen network, whereas the inner layer is represented by an octahedral aluminium–oxygen unit. HNTs have been largely adopted as materials for environmental and catalytic applications; however, they possess a natural inert character,^52–54^ and their surface modification is needed to strengthen the interaction between catalyst and support.^55–57^ Furthermore, the morphology of the catalytic support may influence the diffusion of active metal precursor in the impregnation step.^58^

In the literature, several examples of chemical HNT surface modification methods have been reported. Generally, they can be divided into acid treatment and alkali treatment based on the nature of the used chemical. The ultimate goal of both acid and alkali treatments is to create defects on the HNT surface or to unzip the typical spiral structure of the HNT. A similar approach was applied to different bulk materials (*e.g.*, boron nitride and TiO_2_), as an effective way to expose more active sites and to improve the catalytic activity of those materials.^59–63^ Acid treatment with HNO_3_ has been reported to increase the catalytic performance of nickel on HNT for steam–CO_2_ dual reforming of methane.^55^ A strategy to increase the surface area of HNT was represented by etching with sulphuric acid through the selective etching of Al–OH on the surface of HNT.^64^ In studies using alkali treatment, reacting HNTs with a low concentration of sodium hydroxide (NaOH) resulted in the formation of hydroxyl groups on the surface of HNTs, allowing for a higher dispersion of the treated HNT in water and polar solvents.^65^ An interesting approach towards HNT alkali treatment is represented by the use of molten salts. Such an approach was reported by Chen *et al.* for the fabrication of Pt@HNT catalysts, resulting in improved activity and selectivity for the hydrogenation of cinnamaldehyde.^56^ In a similar fashion, Zhang and his group selectively etched the outer silica layer of halloysite by ball milling with molten salts, leading to the formation of defects, where the resulting Ni-based modified HNT catalyst exhibited enhanced catalytic performance, compared to the grinding method.^66^

Over the past decade, various treatment methods have been applied to HNT supports for catalytic methane conversion reactions. In this work, we present a comprehensive comparative study on the effect of acid *versus* alkali treatment on raw halloysite clay together with the catalytic activities of the corresponding nickel-based catalysts. Specifically, raw HNT (S1) was modified by treatment with HNO_3_ (S3), H_2_SO_4_ (S5), ball-milled molten salt (S7), NaOH (S9), and ground molten salt (S11), and its textural properties were investigated. The six HNT samples were doped with nickel salt using the wet impregnation method. The resulting doped catalysts S2 (Ni@S1), S4 (Ni@S3), S6 (Ni@S5), S8 (Ni@S7), S10 (Ni@S9), and S12 (Ni@S11) were extensively characterized and tested in a DRM reaction. Various characterization techniques, including TEM, BET, ssNMR, XRD, CO_2_-TPD, TGA, and H_2_-TPR, were employed to investigate the effects of the different support treatments. Our findings indicate that the activity and stability of the final catalysts were strongly affected by the type of treatment owing to different Ni dispersion, reducibility, basicity, and different numbers of active sites. In general, alkali treatment of the HNT support yielded more stable catalysts in the DRM, with an overall higher CH_4_ and CO_2_ conversion than those recorded for supports treated with acids.

## 2. Methodology

### 2.1 Catalyst preparation

A series of Ni-based halloysite catalysts were synthesized by acid treatment (HNO_3_ and H_2_SO_4_) and by alkali treatment (NaOH and Na_2_CO_3_–NaNO_3_ molten salt), followed by wet impregnation. Nano-halloysite clay (Al_2_Si_2_O_5_(OH)_4_·2H_2_O) was purchased from Sigma-Aldrich and used as support. Nitric acid (HNO_3_ ≥69.0%), sulphuric acid (H_2_SO_4_ 95–97%), nickel chloride hexahydrate (NiCl_2_·6H_2_O), sodium hydroxide pellets (NaOH), sodium nitrate (Na_2_NO_3_), and sodium carbonate (Na_2_CO_3_) were all supplied from Sigma-Aldrich and used in the catalyst synthesis process.

The main HNT treatment procedures were adapted from the literature. Specifically, for the acid HNO_3_ (ref. 67) and H_2_SO_4_ (ref. 64) treatments, halloysite (2 g) was refluxed in 120 mL of 3 M HNO_3_ or H_2_SO_4_ solution for 8 hours. The resulting solid was washed several times with deionized (DI) water until it reached pH 4 to 5; the samples were then dried at 105 °C for 12 hours, followed by calcination at 1000 °C for 6 hours. In the alkali (NaOH) treatment, halloysite (10 g) was mixed in 100 mL of 5 M NaOH solution and heated to reflux for 8 hours. The obtained solid was then washed several times with DI water until a pH of 6 to 7 was achieved; the samples were then dried at 105 °C for 12 hours.^65^ For the molten salt treatment of HNT nanotubes, a mixture of HNT (2 g), Na_2_CO_3_ (0.6 g) and NaNO_3_ (2 g) was milled in a ceramic ball mill device at a speed of 300 rpm (ball-to-sample mass ratio ≃ 10 : 1) for an hour and then calcined at 350 °C for 2 h. Next, the resultant sample was washed several times with deionized water to remove soluble salts and impurities and then dried in a vacuum oven for 12 hours. The effect of grinding the solid mixture rather than using ball milling was also explored.^66^ Raw halloysite nanotubes were used as a reference to evaluate the effect of the treatments.

The impregnation method was used to synthesize Ni-based halloysite nanotubes. For this purpose, a certain amount of nickel chloride, with a nominal content of 10%, was dissolved in deionized water, after which the treated HNT nanotubes were added to the prepared aqueous solution at room temperature. The obtained slurry was accurately mixed for 1 hour, after which urea was added to the mixture in excess with respect to nickel salt weight. The mixture was stirred for a further 60 minutes at room temperature prior to refluxing at 80 °C for 10 hours. In the next step, the resulting solid was washed and dried in an oven for 12 hours. Finally, all the samples were calcined at 800 °C for 6 hours. Throughout this paper, the samples were labelled as follows: raw HNT (S1); Ni@raw HNT (S2); HNO_3_-HNT (S3); Ni@HNO_3_-HNT (S4); H_2_SO_4_-HNT (S5); Ni@H_2_SO_4_-HNT (S6); ball-milled molten salt-HNT (S7); Ni@ball-milled molten salt-HNT (S8); NaOH-HNT (S9); Ni@NaOH-HNT (S10); ground molten salt-HNT (S11); and Ni@grinded molten salt-HNT (S12).

### 2.2 Catalyst characterization

All solid-state NMR experiments were carried out at 14.1 T using a Bruker AVANCE III 600 MHz wide bore spectrometer operating at Larmor frequencies of 600.13 MHz for ^1^H and 119.2 MHz for ^29^Si, equipped with a Bruker 3.2 mm triple-resonance low-temperature magic angle spinning (LT-MAS) NMR probe. Samples were ground into fine powders and packed into 3.2 mm outer diameter zirconia rotors. The ^29^Si CP-MAS (cross-polarization magic angle spinning) spectra were acquired at room temperature with a 10 kHz MAS rate using a ramped-amplitude cross-polarization pulse sequence with a ^1^H π/2 excitation pulse of 3.0 μs, a recycle delay of 3 s, and cross-polarization contact times ranging from 5.0 to 8.0 ms. SPINAL-64 heteronuclear decoupling with *ca.* 90–100 kHz RF field strength was applied to decouple protons during signal acquisition,^68^ along with the collection ranging from 1280 to 2048 FIDs to obtain reliable signal-to-noise ratios. Additionally, ^29^Si single pulse (SP) experiments with high-power proton decoupling were performed on some samples at a 10 kHz MAS rate using a 1.5 μs (π/6 flip angle) ^29^Si excitation pulse and a 15 s recycle delay with the accumulation ranging from 1120 to 2000 transients. All ^29^Si NMR spectra were externally referenced with respect to liquid tetramethylsilane (TMS) at 0 ppm.

Specific BET surface areas of the as-prepared catalysts were measured by a surface area analyser (ASAP 2420, Micromeritics) at 77 K. Before measurements, samples were dried at 90 °C for 30 min, followed by degassing at 350 °C for 6 h under vacuum to remove contaminates and water. The specific surface area was calculated using the BET equation and BJH desorption for pore size analysis.

The X-ray powder diffraction (XRD) patterns of the prepared catalysts were conducted using an X-ray diffractor (XRD-6100x, Shimadzu) with Cu-Kα (*λ* = 1.5406 Å) radiation. The measurement was conditioned at 40 kV and 30 mA, with a scanning speed of 7° min^−1^ ranging from 10° to 70°.

The TEM investigations were performed on a field emission gun TALOS (FEI, Hillsboro, Oregon, USA) operated at 200 kV and equipped with an FEI Energy Dispersive X-ray (EDX) detector and a high-angle annular dark-field, HAADF detector. Esprit software from Bruker (Billerica, Massachusetts, USA) was used to obtain the qualitative element analyses. Specimens were prepared by dispersing samples in isopropanol and droplets of the suspension were deposited on a thin copper grid. After the isopropanol was vaporized, the thin copper grid was mounted in a TEM holder and then inserted into the sample chamber of the transmission electron microscope. Moreover, actual Ni contents on the as-prepared catalysts were performed by SEM-EDS FEI Quanta650FEG for imaging and equipped with a Bruker XFlash 6I60 detector for EDS analysis.

H_2_ temperature-programmed reduction (H_2_-TPR) of the as-prepared catalysts was performed by applying an AutoChem 2950 (Micromeritics) apparatus equipped with thermal conductivity. Prior to the experiments, 50 mg of catalyst was pretreated in N_2_ (100 mL min^−1^) at 300 °C for 30 min to remove impurities, followed by cooling to ambient temperature in N_2_ stream. Catalysts were reduced with a 10 vol% H_2_–Ar mixture (100 mL min^−1^) by heating up to 800 °C, at a ramp rate of 10 °C min^−1^.

Catalyst basic sites were identified through CO_2_-temperature programmed desorption (CO_2_-TPD) analysis using AutoChem 2950 apparatus, where 500 mg of catalyst was pretreated in N_2_ (100 mL min^−1^) at 300 °C for 30 min to remove impurities. After cooling to ambient temperature in N_2_, the catalyst was saturated with CO_2_ gas (100 mL min^−1^), followed by flushing the sample with Ar (100 mL min^−1^) to remove excess CO_2_. Finally, samples were heated up to 800 °C at a ramp rate of 10 °C min^−1^ while detecting the CO_2_-desorption.

Pulse chemisorption was also performed by an AutoChemII 2950 station. The nickel-doped samples (S2, S4, S6, S8, S10, and S12) were placed in a U-shaped stainless reactor to quantify the Ni-dispersion. Prior to the experiments, 50 mg of catalyst was reduced by 10 vol% H_2_–Ar mixture (50 mL min^−1^) and heated up to 750 °C, at a ramp rate of 10 °C min^−1^. Then, Ar gas was purged to remove the excess H_2_, at 775 °C for 60 minutes, followed by cooling down to ambient temperature in Ar. Pulse chemisorption was performed at 35 °C, where a cryo-cooler from micromeritics was used to inject liquid nitrogen to cool and maintain the sample at the desired temperature. The volume of the injection loop was 0.5 cm^3^. Pulses of H_2_ (10 vol% H_2_ in Ar) were injected into the catalytic reactor, and Argos was used as the carrier gas. H_2_ consumption, TPR and CO_2_-TPD were all measured using a thermal conductivity TCD detector, equipped with a water trap. Pulse injection, sample temperature and TCD signals were controlled and monitored by Micromeritics AutoChem software.

Thermogravimetric analysis (TGA) of both the reduced and spent catalysts was performed using a Discovery TGA from TA Instruments. The catalysts (∼10 mg) were heated up to 800 °C at a rate of 10 °C min^−1^ with air flow (20 mL min^−1^).

### 2.3 Catalytic activity and stability tests

Catalytic activity measurements of the DRM were performed using a MicroEffi reactor (EMP) equipped with an SS310 fixed-bed reactor (internal diameter 9.1 mm and length 304.8 mm). Approximately 200 mg of catalyst was loaded and reduced by 5% H_2_/Ar (100 mL min^−1^) at 800 °C. The reactor was then cooled to 750 °C by N_2_ flow (50 mL min^−1^). A mixture of equimolar CH_4_/CO_2_ (50 mL min^−1^) passed through the catalyst with a gas hourly space velocity (GHSV) of 30 000 mL g^−1^ h^−1^. Catalytic performance was evaluated at 750 °C for 24 hours. Gaseous products (*e.g.*, CO, H_2_, CH_4_ and CO_2_) were analysed by applying an on-line mass spectrometer mks-CIRRUS™ 2 atmospheric pressure gas monitoring. Conversions of CH_4_ and CO_2_, as well as the molar ratio of H_2_/CO, were determined from the corresponding difference between the outlet and inlet flow rates. The CH_4_ and CO_2_ conversions (%) as well as H_2_/CO molar ratio were calculated using the following equations:
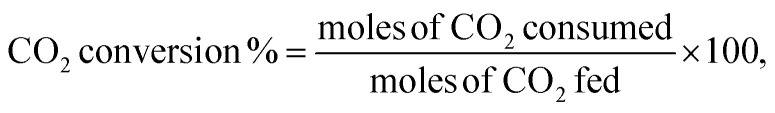

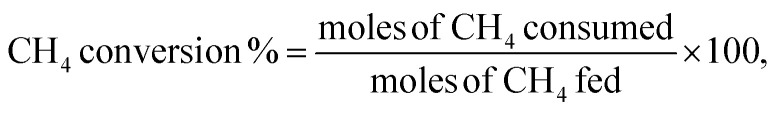

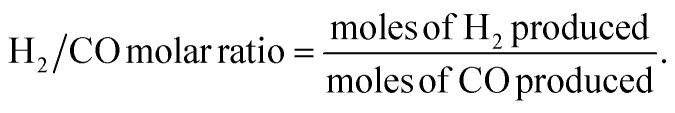


## 3. Results and discussion

Aluminosilicate nanotubular clay is selected because of its low cost, natural availability, chemical/thermal stability and relatively high surface area (approx. 60–80 m^2^ g^−1^).^51,69–71^ The acid treatment of HNT is a common strategy used by the materials community that involves the use of strong acids, such as sulfuric acid or nitric acid, to modify the surface of the nanotubes.^64,72,73^ This process leads to the removal of impurities and amorphous materials, resulting in the exposure of a higher proportion of the tubular structure, by etching the inner alumina layer (and in a minor percentage the silica layer) and thus enhancing the material's ability to interact with the metal catalyst. The acid treatment process also affects the porosity and active sites on the surface of the HNT, making it more accessible for reactant molecules to interact with the substrate. These pores and active sites provide sites for catalysis, increasing the material's reactivity and efficiency. The availability of pores and active sites upon acid treatment makes HNT a more effective adsorbent and catalyst support. This allows for higher loading of active species and facilitates the diffusion of reactants into the inner structure of the nanotubes.

Recently, alkali-treated HNTs have attracted wide attention as a valuable alternative to acid-treated HNT.^65,74,75^ Alkali treatment involves the use of alkaline solutions, such as sodium hydroxide (NaOH) or Na_2_CO_3_–NaNO_3_ molten salts, to modify the surface of the HNT. This process can create different functional groups (*e.g.*, –OH) on the nanotube surface, which can enhance its interaction with carbon dioxide (CO_2_) molecules, leading to a higher CO_2_ adsorption capacity. Furthermore, alkaline treatment can increase the resistance of HNT to coke deposition, which is the accumulation of carbonaceous materials on the catalyst surface during reactions, prolonging the catalyst's lifespan and maintaining its activity over extended reaction periods. Additionally, alkali treatment may unzip the tubular structure of the HNT, exposing more of its surface.

Both acid and alkaline treatments of HNT offer distinct advantages. Acid treatment increases the surface area and availability of pores and active sites, while alkaline treatment enhances CO_2_ adsorption, resistance to coke deposition, catalyst activity, and stability. These treatments make HNT a versatile material with valuable applications in adsorption and catalysis. The choice between acid and alkaline treatments depends on the specific needs and intended applications of the modified HNT.

### 3.1 Solid-state NMR analysis

**Fig. 1:**
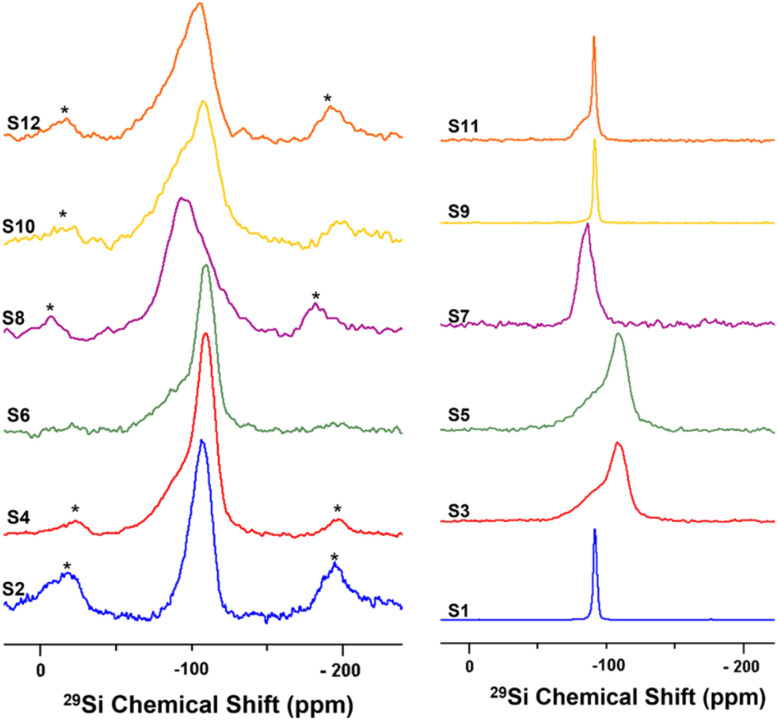
Solid-state ^29^Si NMR spectra at 10 kHz MAS for undoped samples (right) and nickel-doped catalysts (left).

Solid-state nuclear magnetic resonance (ssNMR) spectroscopy was used to better demonstrate the effects of the different treatments on the silica outer layer of HNT, and the spectra are shown in Fig. 1. The ^29^Si CP-MAS NMR spectrum of raw HNT (S1) shows a single sharp signal at −91.8 ppm (from TMS) corresponding to the crystallographically equivalent Si atoms of the tetrahedral SiO_4_ layers on the HNT outer surface in accordance with reported values in the literature.^76^ Moreover, the observed chemical shift indicates a Q^3^-type halloysite silicon site, Si(OSi)_3_(OAl_2_), in which each silicon atom of the SiO_4_ tetrahedron is bridged by one oxygen atom to two aluminium atoms from the adjacent AlO_6_ octahedral layer in its second coordination sphere.^76–78^ It is worth noting that the Q^*n*^ assignments used herein are consistent with the notations used by Lippmaa *et al.* and Smith *et al.* for silicates and layered aluminosilicates.^79,80^ According to this notation, all silicate anions can be described as a combination of Q^*n*^ units, where the Q symbol represents a silicon atom bonded to four oxygen atoms (Q = SiO_4_) and the superscript shows the number of other Q units (Si–O tetrahedra) attached to the silicon tetrahedron under study.

For S3, a broad silicon signal centred at −109 ppm is observed; this peak can be attributed to the formation of a Q^4^-type Si(OSi)_4_ amorphous three-dimensional silica site as a result of the destruction of the HNT crystal lattice by acid treatment. The downfield (high-frequency) shoulder might be attributed to some silicon-rich nanoparticles or minor Q^3^-type sites from unreacted HNT. Similarly, S5 shows a broad silicon signal at −110 ppm. Ni-doped catalysts S2, S4, and S6 show a broad silicon signal at −110 ppm, similar to the acid-treated HNTs (S3 and S5, respectively), attributed to the common calcination step after doping. The HNT treated using the ball-milled molten salt method (S7) shows a broad silicon signal at −86.6 ppm. This signal could be assigned to Q^2^-type silicon environments, such as Si(OSi)_2_(OH)_2_ or Si(OSi)_2_(OH)(OAl) sites. Interestingly, its Ni-doped counterpart, S8, shows a broad and featureless silicon signal centred around −93 ppm. S9 shows a silicon signal identical to that of the unreacted raw HNT at −92 ppm. Here, it is important to highlight that NaOH treatment is the only method not comprising a calcination or heating step at the end (other than the drying step). In contrast, S10 shows a broad featureless silicon signal centred at −108 ppm. S11 shows a signal at −91.3 ppm, similar to the one in S1, and a downfield shoulder that might correspond to minor Q^2^-type environments, such as Si(OSi)_2_(OH)_2_ or Si(OSi)_2_(OH)(OAl), suggesting the partial efficacy of the treatment and the presence of unreacted raw HNTs. Instead, S12 shows a broad and featureless silicon peak centred at −106 ppm. In general, the broad and almost featureless ^29^Si NMR line shapes observed for some of the samples are likely due to the presence of disordered and/or amorphous electronic environments around the silicon atoms in these solids as a consequence of both chemical and thermal treatments. This structural disorder is caused by variations in local geometry around a certain nucleus, such as changes in the number and type of coordinating atoms, the nature of atoms on the next nearest neighbouring sites, and variations in bond lengths and angles. From an NMR perspective, it has been demonstrated that such a disorder in the structure around a nucleus causes a distribution in the NMR chemical shift of that nucleus, which results in such broadened line shapes.^81^

### 3.2 XRD investigation

**Fig. 2:**
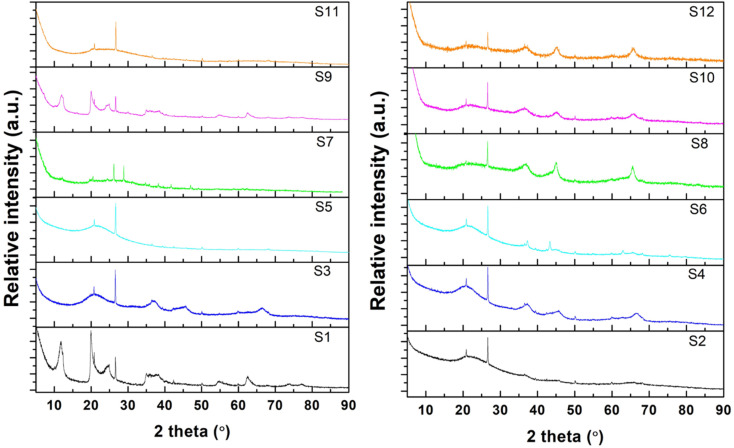
XRD patterns of the synthesized HNTs: undoped samples (left) and impregnated samples (right).

The XRD patterns of all samples are shown in Fig. 2. For raw halloysite (S1), the XRD pattern showed the characteristic peaks of pristine halloysite at 2*θ* = 11.78°, 19.9°, 24.8°, 35.2°, 54.5°, 62.1°, and 63.5 according to JCPDS#29-1487.^82^ A small amount of SiO_2_ was also observed, which agrees with the chemical analysis of halloysite.^83^ Amongst the undoped samples, the non-calcinated ones (S1 and S9) retained their typical HNT peaks. However, all the other undoped samples (S3, S5, S7, and S11), whose preparation procedures included a calcination step after treatment, did not show the characteristic peaks of raw halloysite, revealing the formation of amorphous halloysite.^84^ Similarly, for all the impregnated samples, the HNT peaks disappeared after calcination at 800 °C. Furthermore, the relative intensity of the SiO_2_ peak to the structural peaks of the halloysite phase increased for the modified samples, indicating the extraction of silica from the halloysite structure.^67,83,85^ In the impregnated samples, no peaks were observed for NiO, demonstrating an effective reduction to metallic nickel in the presence of urea. Peaks for metallic nickel were observed for samples S6, S8, and S10, and nickel aluminium oxide spinel peaks were observed for S8 only.^67^

### 3.3 Morphology investigation

Scanning electron microscopy and energy-dispersive X-ray spectroscopy (SEM-EDS) were conducted for the doped catalyst to evaluate the amount of loaded nickel. Despite the initial loading of 10 wt% nickel, the final percentage of nickel ranged from 3.6 to 11%, with S10 and S2 having the lowest loadings (see Fig. S1†).

**Fig. 3:**
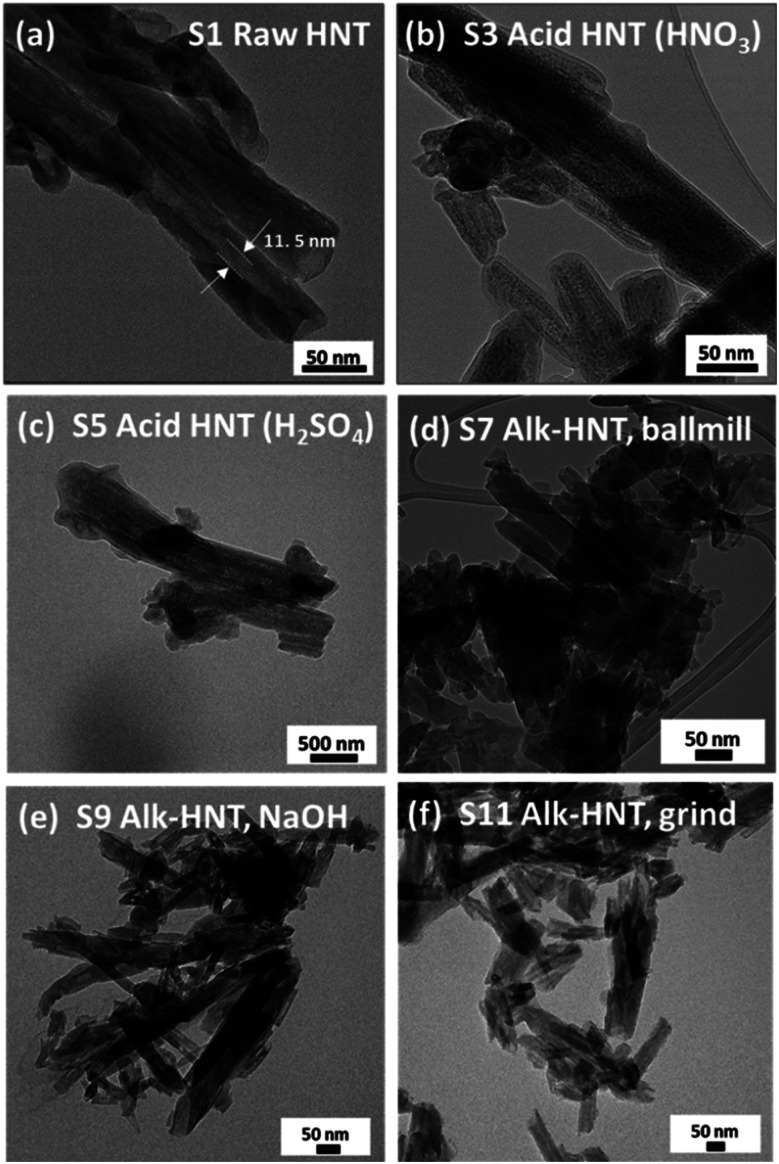
TEM images of the treated supports: (a) S1, (b) S3, (c) S5, (d) S7, (e) S9, and (f) S11.

To better understand the changes in the morphology of the HNTs upon treatment, transmission electron microscopy (TEM) was conducted on the raw HNT support and the treated HNT supports (Fig. 3). Fig. 3a illustrates the structure of the raw HNTs, depicting a nanotubular structure with an inner lumen diameter of about 11.5 nm. Fig. 3b and c show the microscopic images of HNO_3_ and H_2_SO_4_ treated HNT, respectively. Here, the HNT walls and edges appear smoother and more rounded hypothetically owing to acid treatment, whereby the acid attacks the walls of the HNT, particularly the alumina layer, as alumina is more soluble in acid than silica.^64,67^ The etching of alumina *via* acid could also be the reason why the inner lumen area (consisting of alumina) of acid-treated samples is less vivid.

However, HNTs subjected to alkaline treatment with NaOH and the molten salt methods do not show similar smooth edges throughout the HNT, as depicted in Fig. 3d and e, respectively. However, the lumen area is not very visible. Alkaline treatment dissolves silica, which could have caused the collapse of the HNT walls, destroying the lumen area. Specifically, for the HNT treated with the ball-milled molten salt method (Fig. 3e), the HNTs adopt a more plate-like structure rather than their original tubular shape. On the contrary, no such changes are observed in the molten salt grinding method, where the HNTs retain their tubular morphology. In general, all the HNT samples treated with alkalis resulted in tubular shapes that were shorter and smaller in diameter than those of their acid-treated counterparts.

**Fig. 4:**
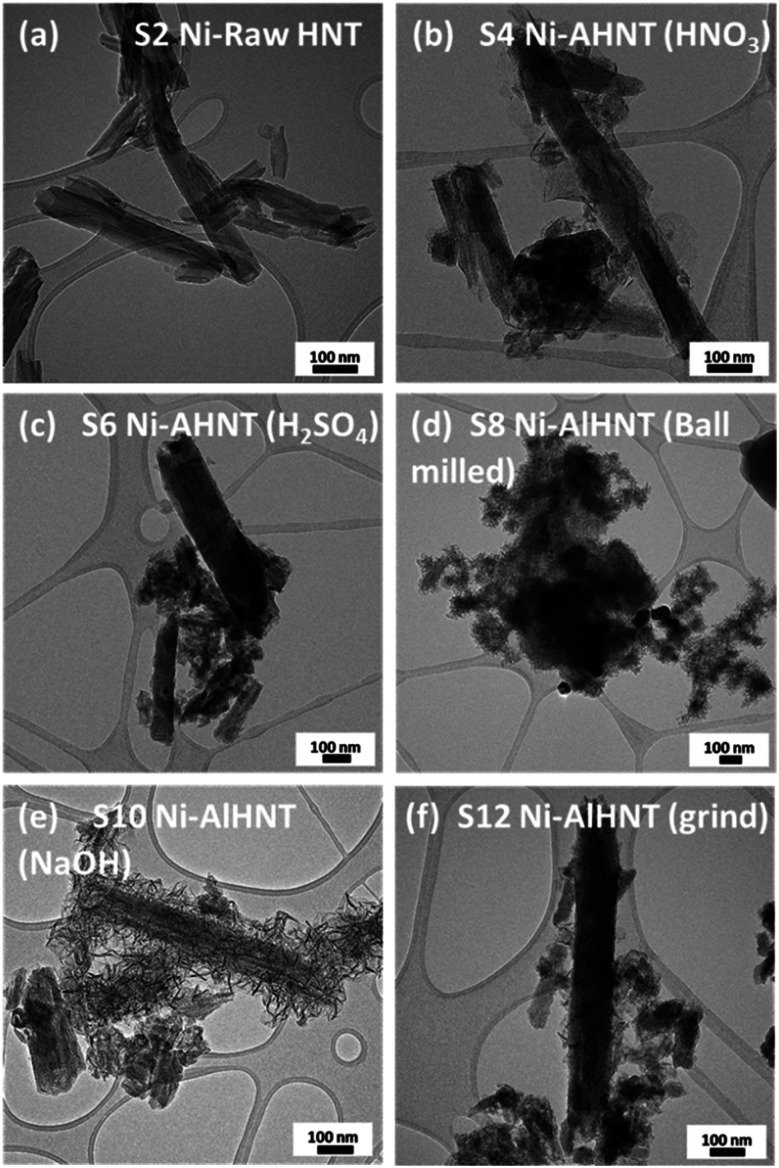
TEM images of Ni loaded on HNT supports: (a) S2, (b) S4, (c) S6, (d) S8, (e) S10, and (f) S12.

TEM characterization was conducted on the Ni metal-doped supports, and the results are presented in Fig. 4. Visible nickel particles were not observed in the TEM image for Ni-raw HNT (Fig. 4a); instead, Ni was found to be uniformly distributed through the support in the STEM-EDX elemental mapping (Fig. S2†). Interestingly, the presence of nanosheets was observed on most of the treated supports with finer nanosheets growing on the alkali-treated supports (Fig. 4d–f). The Ni particle size was calculated using TEM images and image processing software (see Table S2 and Fig. S6†), revealing smaller particles for S2, S4, and S6 in the range of 7–9 nm, and larger particles for the alkali-treated samples S8, S10, and S12, in the range of 13–30 nm.

### 3.4 Textural properties

The nitrogen adsorption–desorption isotherms and pore size analyses of the samples are reported in Fig. S3 and S4,† respectively. All samples showed a type IV isotherm with a hysteresis loop, demonstrating the existence of mesopores according to the IUPAC classification.^86^ The corresponding BET surface areas and pore volumes are summarized in Table S1.† The BET surface area of the alkali-treated samples generally decreased from 65 to 19, 50, and 47 m^2^ g^−1^ (for S7, S9, and S11, respectively), while the total pore volume changed from 0.275 to 0.105, 0.321, and 0.277 cm^3^ g^−1^ (for S7, S9, and S11, respectively), when compared to the raw halloysite, owing to the effect of silica etching. Additionally, in the acid-treated HNT, a general decrease in BET surface area and a similar pore volume, respectively, was observed, for the HNO_3_-treated sample (40 m^2^ g^−1^ and 0.230 cm^3^ g^−1^) and for the H_2_SO_4_-treated sample (62 m^2^ g^−1^ and 0.354 cm^3^ g^−1^) compared to raw HNT. This behaviour can be attributed to the favourable etching of alumina instead of the silica layer when using acid treatment, resulting in better preservation of the original structure, as observed in the TEM images.

After decorating all the samples with Ni, the BET surface area and pore volume of the metal-containing samples were higher than those of the corresponding undoped halloysite samples (see Table 1). Therefore, the resulting pore structure was found to be sufficient to allow for the sufficient diffusion of methane molecules and products.

**Table 1:** Textural properties of the Ni-doped HNTs, BET and pore size analysis

Sample	*S*_BET_ [m^2^ g^−1^]	Micropore area [m^2^ g^−1^]	*V*_*t*_ [cm^3^ g^−1^]	Pore size [nm]	Si/Al ratio
S2: Ni-raw HNT	53	12	0.281	13.9	1
S4: Ni-AHNT, HNO_3_	55	13	0.269	12	1.5
S6: Ni-AHNT, H_2_SO_4_	66	10	0.298	12.4	1.3
S8: Ni-AlHNT, NaNO_3_ ball milled	73.88	5.05	0.290	8.82	0.94
S10: Ni-AlHNT NaOH	50.9	3.14	0.271	16.38	1.03
S12: Ni-AlHNT, NaNO_3_ grinded	52	0	0.244	7.7	1.01

**Fig. 5:**
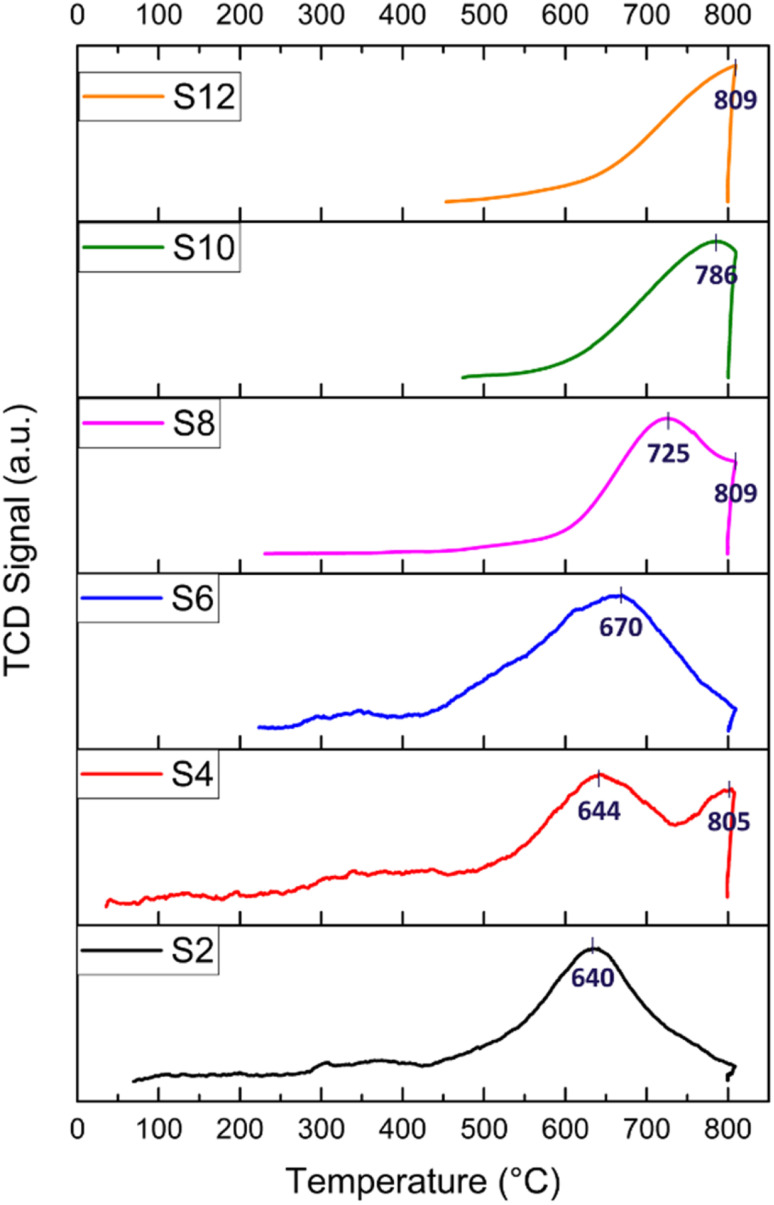
H_2_-TPR profiles of the HNT-supported Ni catalysts; numbers indicate reduction temperatures.

H_2_-TPR experiments were performed to explore the reduction behaviour of the investigated catalysts. H_2_-TPR profiles of the raw and modified halloysite samples are shown in Fig. 5. In general, peaks at temperatures lower than 400 °C indicate the reduction of free NiO. Peaks at temperatures below 600 °C are attributed to metal oxides, such as NiO·Al_2_O_3_ (surface NiAl_2_O_4_ spinel or defeated NiAl_2_O_4_ spinel) or NiO, having a weak interaction with the support. Peaks at temperatures above 600 °C are assigned to the reduction of complex-bonded metal oxide species, such as crystalline NiAl_2_O_4_ spinel, or to the reduction of NiO species strongly interacting with the support.^67,87,88^ Nickel spinels can easily be formed from a reaction between Ni and the Al_2_O_3_ layer. However, it is believed that the NiAl_2_O_4_ spinel cannot be easily reduced to a Ni^0^ species; thus, incomplete reduction of NiAl_2_O_4_ produces Ni sites that interact strongly with the support. Such strong interaction plays an important role in stabilizing the catalytic activity of the Ni during DRM.^88^ Nickel loading for all the prepared catalysts calculated by H_2_-TPR is reported, as shown in Table 2.

**Table 2:** Temperature-programmed desorption (TPD) and reduction (TPR) properties of Ni-doped HNTs

Sample	Ni loading [wt%] from SEM	H_2_-pulse chemisorption		H_2_-TPR			CO_2_-TPD	
		Ni dispersion [%]	Metallic surface area [m^2^ per g metal]	Peaks [°C]	Ni loading [wt%]	H_2_ consumption (cm^3^ per g STP)	Peaks [°C]	CO_2_ adsorption (cm^3^ per g STP)
S2	5.86	0.21	1.38	640	8.82	26.45	73, 394	3.75
S4	7.63	1.29	8.55	644, 805	11.45	34.34	102, 424, 795	8.42
S6	11.01	0.20	1.34	670	11.02	33.07	79	0.09
S8	9.09	0.31	2.03	725, 809	9.85	29.55	86, 401	186.24
S10	3.63	0.74	4.89	786	8.14	24.42	89, 402	555.04
S12	9.57	2.74	18.19	809	6.01%	18.04	77, 395	225.70

For the S2 catalyst, reduction was performed at 640 °C, and the peaks indicated a good interaction between the support and the active metal. Similarly, the acid-treated samples S4 and S6 exhibited a reduction peak at 644 and 670 °C, respectively, in addition to a peak at 805 °C for S4, which could be attributed to the potential dissolution of SiO_2_ by HNO_3_. All the alkali-treated samples (S8, S10, and S12) exhibited reduction peaks at higher temperatures (725, 786, and 809 °C, respectively) compared to the acid-treated catalysts. The reduction of NiO at higher temperatures in the alkali-treated catalysts and in the S4 (805 °C peak) could be attributed to the effect of the alkaline treatment on the morphology of HNT, specifically leading to the formation of sheets (such as with S8) and to the creation of defects on the silica layer. Both two-dimensional nanosheet structures and defect formation strengthened the interaction between NiO and the substrate as well as caused an increased interfacial area. It has been demonstrated that the etching of silica in the outer layer of the halloysite causes some defects and cavities that can confine Ni particles, which, consequently, leads to the formation of a strong metal–support interaction.^18,66^

The H_2_ consumption is calculated for all the catalysts, and it ranges from 18 to 34 cm^3^ per g STP (see Table 2). The highest H_2_ consumption is recorded for S4, which also has the highest Ni loading (11.45 wt%) calculated from the H_2_-TPR. This high Ni loading is responsible for the high initial activity in relation to catalyst performance. Following this, S6 exhibited 33.06 cm^3^ per g STP H_2_ consumption and a Ni loading of 11 wt%, recording the second-best initial conversion. In general, the alkali-treated catalysts exhibited an H_2_ consumption lower than that of the acid-treated samples. In particular, S8 exhibited a reasonable H_2_ consumption of 29.55 cm^3^ per g STP with 9.85 wt% Ni, demonstrating its acceptable catalytic activity with adequate Ni loading owing to the size reduction and good surface area induced by the ball milling process. Both S2 and S10 displayed average H_2_ consumption and Ni loading compared to the other samples, with approximately 8 wt% Ni. Conversely, S12 reported the lowest H_2_ consumption of 18 cm^3^ per g STP, with a total Ni loading of 6 wt%. Notably, S12 underwent a grinding process only, resulting in relatively larger particles compared to the ball-milled technique, thus leading to the lowest final Ni loading among the samples.

Before moving to the CO_2_-TPD analysis, it is important to add a note about the quantification of nickel loading. Despite the nominal 10 wt% loadings discussed in the Catalyst preparation section, the resulting Ni loadings, estimated by both SEM EDS and H_2_-TPR and reported in Table 2, have values that differ from the expected 10%. During the synthesis of the catalyst, the Ni^2+^ ion dissolved in the aqueous media is adsorbed on the surface of the HNT. Based on the previous chemical treatment, the presence of terminal groups, cavities, and other cations, as well as different surface areas, porosity, and dispersion in water, the treated halloysite supports are prone to adsorb nickel ions differently. The resulting reduced nickel catalyst is then estimated by SEM EDS and H_2_-TPR. The values obtained by applying the two methods can be different because the SEM EDS provides a semi-quantitative elemental analysis, and the results are affected by background and matrix effects. Although the metal loading obtained by H_2_-TPR comes from an indirect calculation based on the H_2_ consumption, it is generally considered more accurate than SEM EDS analysis. In general, inductively coupled plasma mass spectrometry (ICP-MS) represents the most accurate technique for measuring catalyst loading on a support, but this analysis is beyond the scope of our work.

**Fig. 6:**
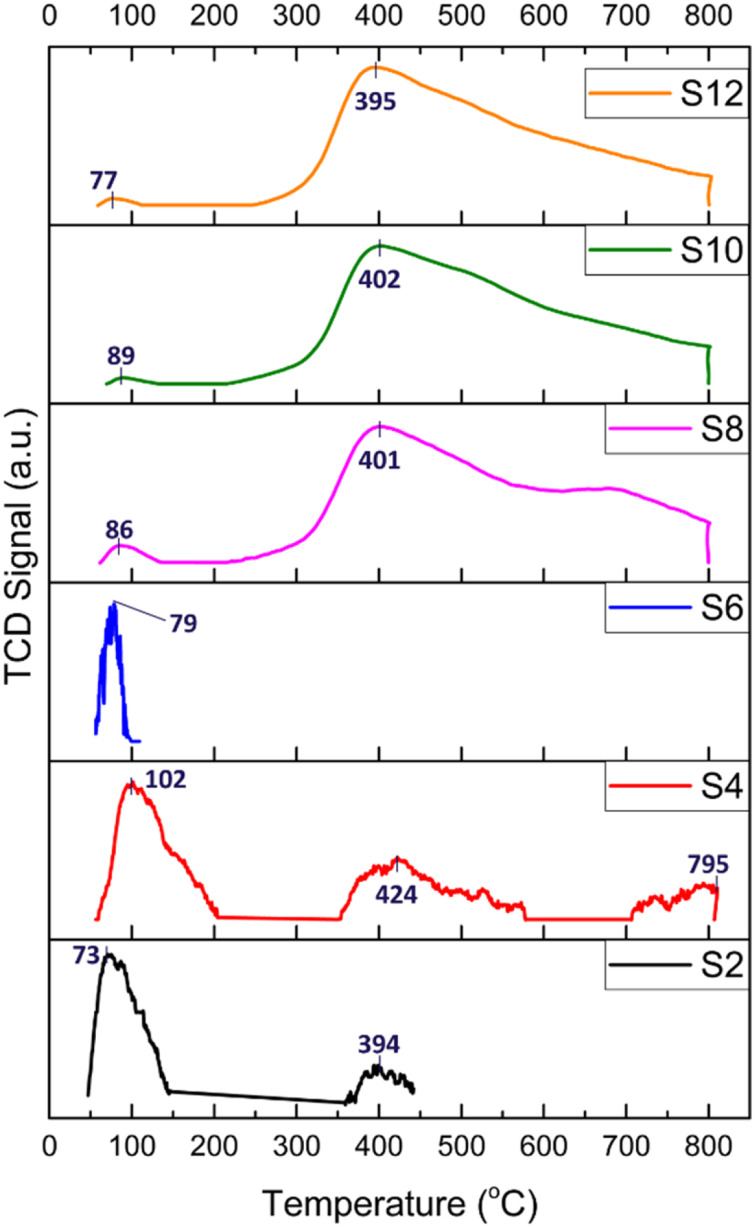
CO_2_-TPD profiles of the HNT-supported Ni catalysts; numbers indicate desorption temperatures.

The basicity of the samples was examined by CO_2_-TPD, and the results are shown in Fig. 6 (the CO_2_ desorption peak values are shown in Table 2). In general, CO_2_ desorption peaks indicate the presence of weak basic sites when *T* < 150 °C (attributed to OH^−^ groups), medium basic sites at *T* = 150–300 °C (attributed to metal–O^2−^ pairs), and strong basic sites when *T* > 300 °C (attributed to isolated O^2−^ anions).^18^

The TPD profiles depicted in Fig. 6 of the acid-treated catalysts (S2, S4, and S6) generally present peaks attributed to a very weak basic strength and a main peak below 102 °C, resulting from reactive bicarbonates sourced from the interactions of CO_2_ with hydroxyl groups on the support surfaces. Interestingly, for S4, three CO_2_ desorption peaks were detected at 102, 424 and 795 °C. This pattern was attributed to the formation of defects and cavities on both the silica and alumina layers of the HNT caused by HNO_3_ treatment, thereby producing a more stable space for the confinement of CO_2_ molecules.

For the alkali-treated catalysts (S8, S10, and S12), the CO_2_ desorption peaks are observed at higher temperatures, which typically indicate a relatively stronger basic site. The desorption peak was around 400 °C and could be linked to more stable carbonates stemming from the interactions of CO_2_ with the surface oxygen atoms of Ni. Moreover, the area of the desorption peaks reflects the number of basic sites on the surface of the catalysts. As depicted in Fig. 6, the area of the desorption peaks of the alkali-treated catalysts was much larger than that of the acid-treated catalysts.

**Fig. 7:**
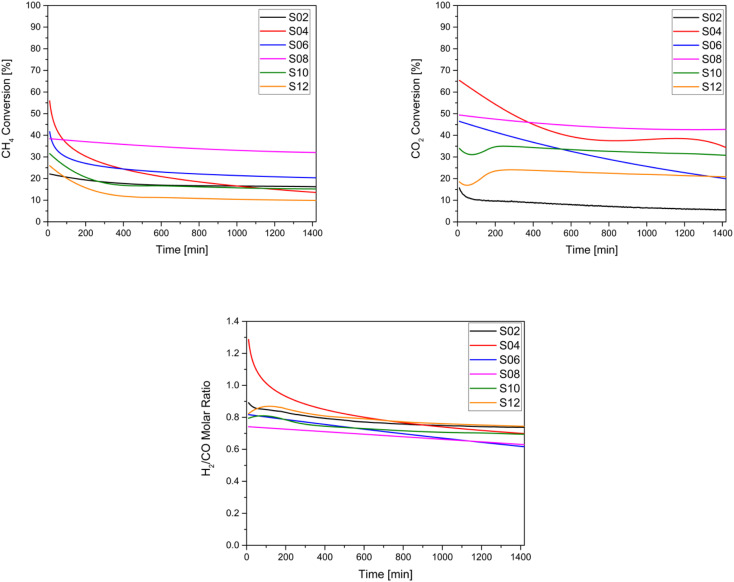
Catalytic performances of the studied catalysts: (a) CH_4_ conversion (b) CO_2_ conversion and (c) H_2_/CO molar ratio.

The CO_2_ consumption of the samples is obtained by CO_2_-TPD, as detailed in Table 2. The raw HNT and acid-treated samples, S2, S4, and S6, exhibited CO_2_ consumptions of 3.75, 8.42, and 0.09 cm^3^ per g STP, respectively. These findings confirm their low basicity and subsequent lack of stability, as observed in the performance test (Fig. 7). In contrast, the alkali-treated samples, S8, S10, and S12, demonstrated significantly higher values at 186.24, 555.04, and 225.70 cm^3^ per g STP, respectively. These results indicate the elevated basicity of these samples owing to the formation of hydroxyl terminations, resulting in higher CO_2_ adsorption, reduced coke formation, and, consequently, a more stable catalyst, as corroborated by the stability performance test.

The Ni dispersion and the corresponding Ni metallic surface area, as derived from the H_2_-pulse chemisorption, are presented in Table 2. The Ni dispersion values range from 0.2% to 2.7%, with corresponding Ni metallic surface areas ranging from 1.3 to 18.2 m^2^ g_Ni_^−1^.

Similar low dispersion values in clay-based catalysts, as observed here, have been commonly reported in the literature. For example, Zhao *et al.* reported comparable values between 0.17 and 0.6 for a similar acid-treated catalyst.^55^ Moreover, the pulse chemisorption experiments suggest that the Ni metallic surface area might not be the sole factor governing the catalytic reaction. In fact, catalyst S12, with the highest Ni surface area of 18.2 m^2^ g_Ni_^−1^, demonstrated poor catalytic performance and stability.

### 3.5 Catalyst activity and stability evaluation

Because DRM is an endothermic reaction, based on the Gibbs free energy, it requires a temperature in the range of 627–1000 °C and atmospheric pressure to approach equilibrium conversion, a reduction of carbon deposition, and stability.^89^ Here, the catalytic activity of the samples was evaluated at a temperature of 750 °C. For these tests, the conversion of CH_4_ and CO_2_ as well as the measurement of the H_2_/CO ratio were conducted. As illustrated in Fig. 7, it can be observed that CO_2_ conversion was generally higher than CH_4_ conversion for all samples, except for the untreated Ni-HNT (S2) and for S6 owing to their low basicity. Thus, the reverse water-gas shift reaction becomes favourable according to the following equation: CO_2_ + H_2_ ↔ H_2_O + CO. Amongst the catalysts, the overall highest CO_2_ and CH_4_ conversions were observed for S8 (ball-milled molten salt method), which also depicted remarkable stability over 24 hours of testing and was associated with negligible coke deposition. However, S12 (ground molten salt method) depicted the lowest CH_4_ conversion (10%) and the second lowest CO_2_ conversion (12%) after S2 owing to the lowest Ni loading (6 wt%, see Table 2) and potentially in the absence of micropores (see Table 1), which leads to a shorter residence time for the reactants. In general, all the treated catalysts outperformed the CO_2_ conversion recorded for untreated HNT S2. All the analysed catalysts resulted in an average value of 0.75 in terms of the H_2_/CO ratio (Fig. 7), as a consequence of the reverse water–gas shift reaction.

According to Fig. 7, which presents the stability of the samples indicated by the decrease in CH_4_ and CO_2_ conversion, no apparent deactivation of S2 and S8 over a period of 24 hours was observed, as the catalysts maintained a stable CH_4_ and CO_2_ conversion. In contrast, a significant deactivation was largely observed for S4 and S6 (both acid treated), where the conversion dropped from 56% to 14% (CH_4_) and 65% to 34% (CO_2_) for S4, and from 42% to 20% (CH_4_) and 46% to 20% (CO_2_) for S6, after 24 h. The high initial conversion exhibited by the acid-treated samples S4 and S6 can be attributed to their relatively higher H_2_ consumption indicated by the H_2_-TPR, along with their overall higher Ni content. Conversely, samples S2 and S12, with lower Ni loading, showed the opposite trend. In general, samples with higher basicity, namely S8, S10, and S12, demonstrated significantly higher CO_2_ adsorption (see Table 2). Thus, they exhibited a negligible CO_2_ conversion drop (see Table 3), corresponding to −7%, −7%, and −0.04%, respectively. To summarise, all the alkali-treated materials appeared to be stable and superior to those supports treated with acids.

**Table 3:** Catalyst performance assessment

Sample	CH_4_ conversion [%]		CO_2_ conversion [%]		Coke formation [%]	Average Ni particle size increases owing to sintering [nm]
	*t* = 10 [min]	*t* = 1440 [min]	*t* = 10 [min]	*t* = 1440 [min]		
S2	22.22	15.83	16.4	6.36	4.71	+42
S4	55.90	13.55	65.39	34.31	33.38	+22
S6	41.68	20.32	46.47	19.96	21.45	+33
S8	38.42	32.02	49.39	42.74	2.67	+17
S10	33.93	15.58	37.77	30.55	13.30	+29
S12	25.89	9.98	21.58	21.54	11.72	+54

The performance testing also revealed that simple mechanical processes, such as ball-milling and grinding, have a strong influence on the textural properties and catalytic conversion of the final catalysts. In this regard, S8 (ball-milling process) outperformed S12 (grinding process) in terms of CO_2_ and CH_4_ conversion, and coke formation. In the future, the modification of catalytic supports, such as halloysites, should not be limited to only chemical treatments but also mechanical processes will certainly play a role in improving the catalytic performances.

### 3.6 Characterization of the spent catalysts

**Fig. 8:**
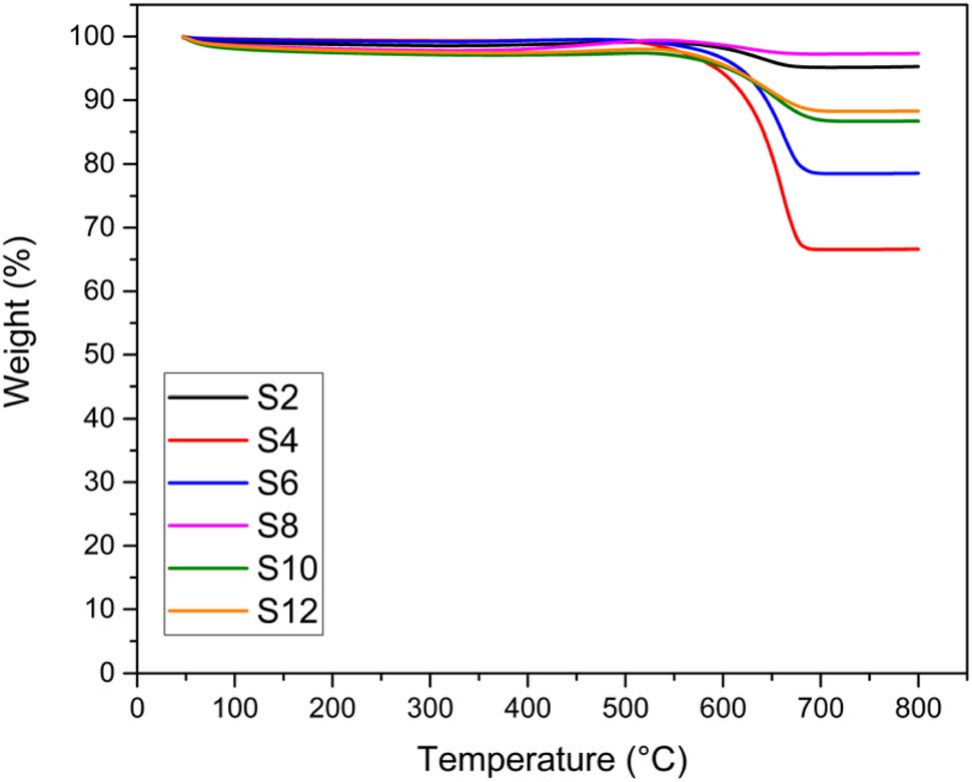
TGA analysis of the spent catalysts.

**Fig. 9:**
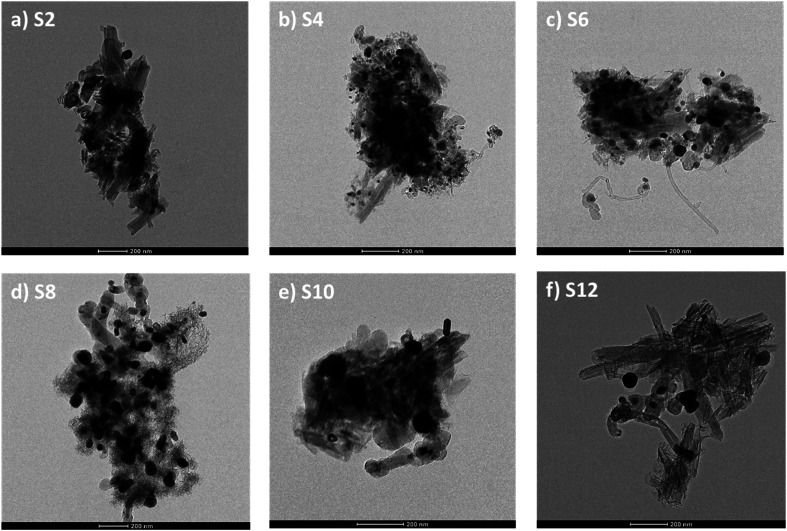
TEM images of spent catalysts: (a) S2 – Ni-raw HNT, (b) S4 – Ni-AHNT (HNO_3_), (c) S6 – Ni-AHNT (H_2_SO_4_), (d) S8 – Ni-AlHNT (ball milled), (e) S10 – Ni-AlHNT (NaOH), and (f) S12 – Ni-AlHNT (ground).

TGA and TEM analyses were performed to investigate the type and quantity of coke deposited on the catalysts, and the results are shown in Fig. 8 and 9, respectively. The weight % from the TGA analysis of the spent catalysts after 24 hours of DRM reaction at 750 °C is also reported in Table 3 in terms of coke formation %. No significant weight loss (>5%) was recorded for the fresh samples (Fig. S8†). For all the spent catalysts, only one significant drop in weight % was observed in all TGA profiles in the temperature range of 500–650 °C, indicating only one main type of carbon coke morphology deposited on the catalyst. In general, the number of weight losses in the TGA profile indicates the type of carbon, while the intensity of these signals shows the amount of formed coke. The temperature range at which the main loss occurs is associated with the temperature at which filamentous carbon forms. The weight loss percentages depicted in Fig. 8 are consistent with the stability exhibited by the catalysts in terms of CH_4_ and CO_2_ conversion drops. Samples showing negligible deactivation over 24 hours of time-on-stream are also those presenting the least coke formation, as highlighted by the minimum weight loss in the TGA profile (*e.g.*, S2 and S8).

TEM images (Fig. 9) of the spent catalysts revealed the formation of carbon filaments. Such filaments gradually accumulate on the support surface by the transformation of active surface carbons within the reaction time, eventually leading to catalyst deactivation. From the figures, it is difficult to estimate the amount of coke formed. However, the general trend seems more in favour of alkali-treated catalysts that show a lower amount of carbon deposited. Interestingly, the acid-treated samples (S4 and S6), with the presence of carbon filaments, also exhibit some amorphous carbon formations. This can also explain the drop in the corresponding TGA profile (Fig. 8) occurring at a temperature lower than that of the other catalysts.

TEM analysis also revealed a typical sintering effect on nickel (Table 3 and Fig. 9). All the spent catalysts showed the formation of large nickel nanoparticles up to 130 nm (Fig. S5†). From the corresponding particle size distribution (Table S2 and Fig. S7†), it can be observed that the mean particle size for spent catalysts ranged from 30 to 67 nm, with the alkali-treated samples characterized by a broader distribution.

The average Ni particle size analysis reveals that although S8 had the highest Ni particle size among the fresh samples, it exhibited the lowest nickel sintering, followed by S10. Conversely, the acid-treated samples S4 and S6, followed by the alkali ground sample, S12, showed a higher Ni sintering effect with respect to the original particle size. This further confirmed that grinding the HNT support after the molten salt treatment did not result in the desired effect otherwise demonstrated by the ball-milling process, which yielded a catalyst with superior resistance against coke formation and sintering. As expected, the raw HNT support reported the highest Ni sintering.

## 4. Conclusions

This work explored the use of aluminosilicate nanotubular clay for catalytic applications, with a focus on the dry reforming of methane. The study highlights the effect of different acid and alkali treatments on HNTs, revealing how these treatment methods affect the material's structure and properties. The reported results showed an enhanced surface area and more accessible active sites in acid-treated HNTs, resulting in higher Ni loading with a smaller particle size, which makes them versatile catalyst supports with higher initial activity. Moreover, alkali-treated HNTs exhibited enhanced CO_2_ adsorption capacity, resistance to coke deposition and to Ni sintering, and overall catalyst stability. This study employed various analytical techniques to characterize the treated HNTs, shedding light on their structural changes. The research also assesses their textural properties, reduction behaviour, basicity, and catalytic performance in DRM reactions. Ultimately, this work provides valuable insights into the potential applications of aluminosilicate nanotubular clay in catalysis and underlines the significance of treatment methods in tailoring its performance for specific applications.

## Conflicts of interest

There are no conflicts of interest to declare.

## Supplementary Material

RA-014-D3RA07990B-s001
