# Supplementary material for: Assessing the effect of acid and alkali treatment on a halloysite-based catalyst for dry reforming of methane
Source: RSC Adv. 2024 Feb 5;14(7):4788–803. doi: 10.1039/d3ra07990b (PMC10840390; doi:10.1039/d3ra07990b)
Supplement: RA-014-D3RA07990B-s001 [file RA-014-D3RA07990B-s001.pdf]

Electronic Supplementary Material (ESI) for RSC Advances.
This journal is © The Royal Society of Chemistry 2024

Supporting information

Assessing the Effect of Acid and Alkali Treatment on Halloysite-Based Catalyst for Dry Reforming of Methane

Ahmed Abotaleb[1], Dema Al-Masri[1,2], Alaa Alkhateb[1], Kamal Mroue[3], Atef Zekri[3], Yasmeen Mashhor[4], Alessandro Sinopoli[1*]

[1] *Qatar Environment and Energy Research Institute, Hamad Bin Khalifa University, P.O. Box 34110, Doha, Qatar*

[2] *Earthna Center for a Sustainable Future, Qatar Foundation, Doha, Qatar*

[3] *HBKU Core Labs, Hamad Bin Khalifa University, P.O. Box 34110, Doha, Qatar*

[4] *Department of Chemistry and Earth Sciences, College of Arts and Sciences, Qatar University, Doha P.O. Box 2713, Qatar*

[*] *E-mail: asinopoli@hbku.edu.qa*

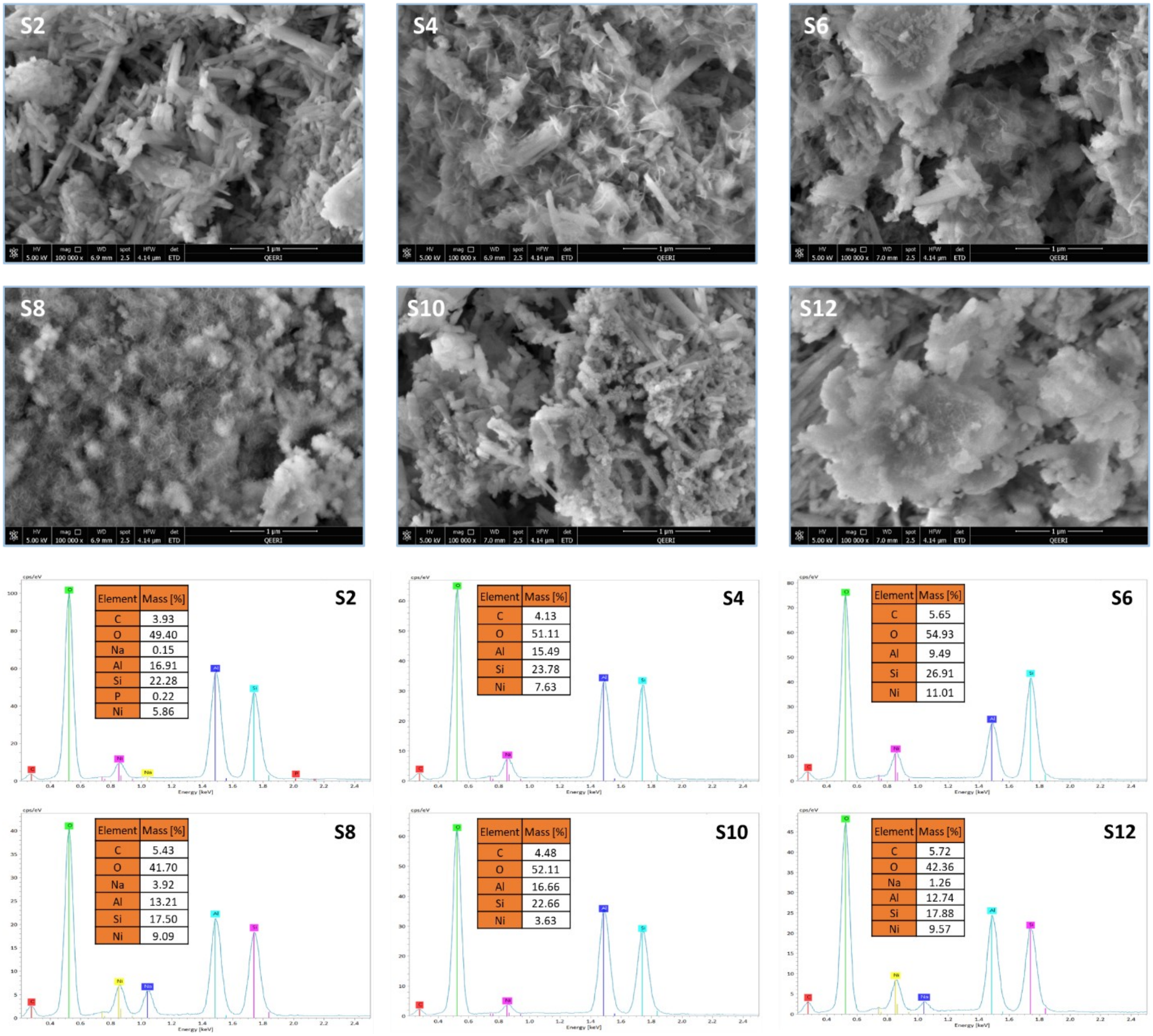

Figure S1. SEM-EDS images with corresponding elemental analysis of (S2) Ni-Raw HNT, (S4) Ni-AHNT (HNO_3), (S6) Ni-AHNT (H_2SO_4), (S8) Ni-AlHNT (ball milled), (S10) Ni-AlHNT (NaOH), and (S12) Ni-AlHNT (grinded).

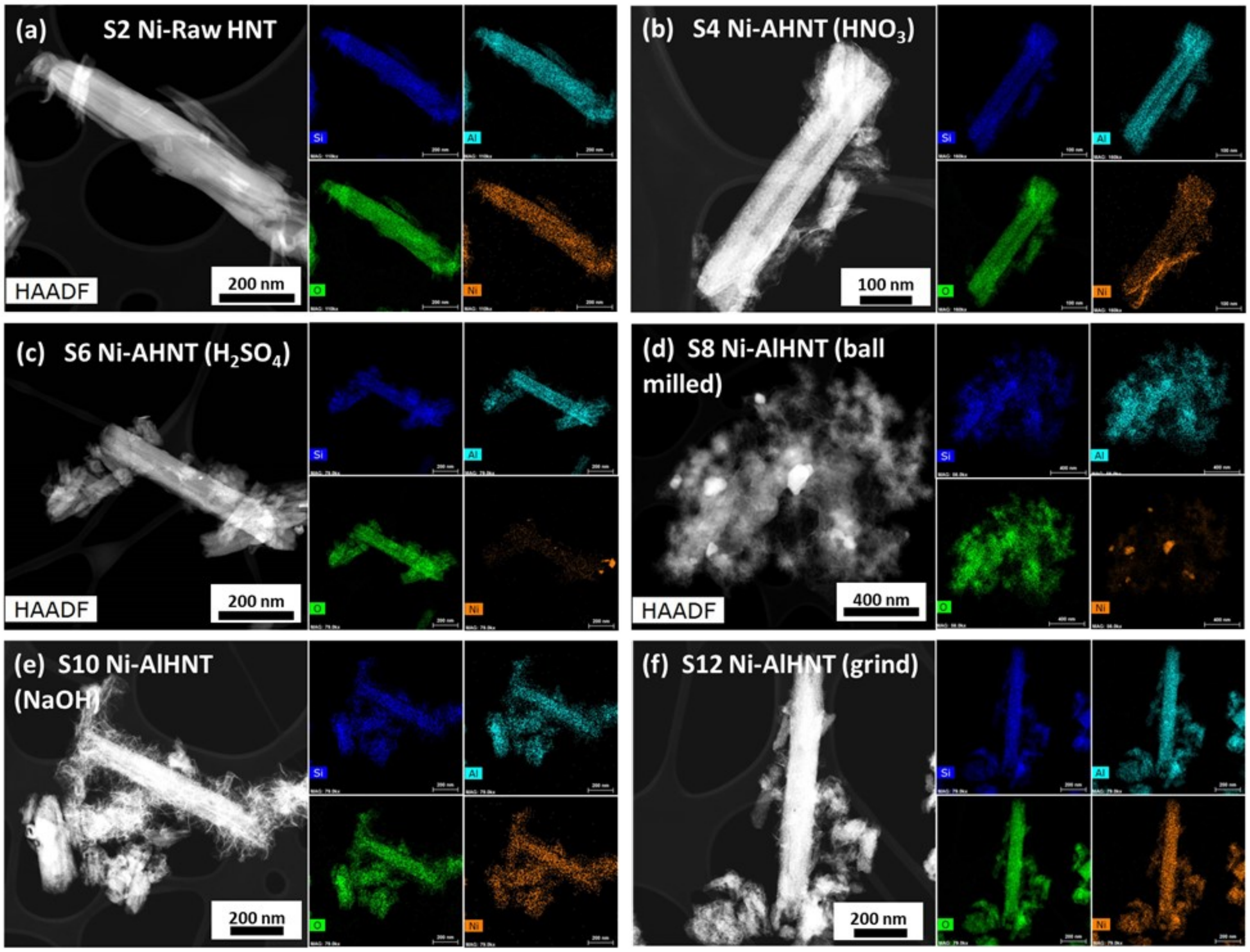

Figure S2. TEM-EDS elemental mapping of Si, Al, O, and Ni on (a) Ni-Raw HNT, (b) Ni-AHNT (HNO_3), (c) Ni-AHNT (H_2SO_4), (d) Ni-AlHNT (ball milled), (e) Ni-AlHNT (NaOH), and (f) Ni-AlHNT (grinded).

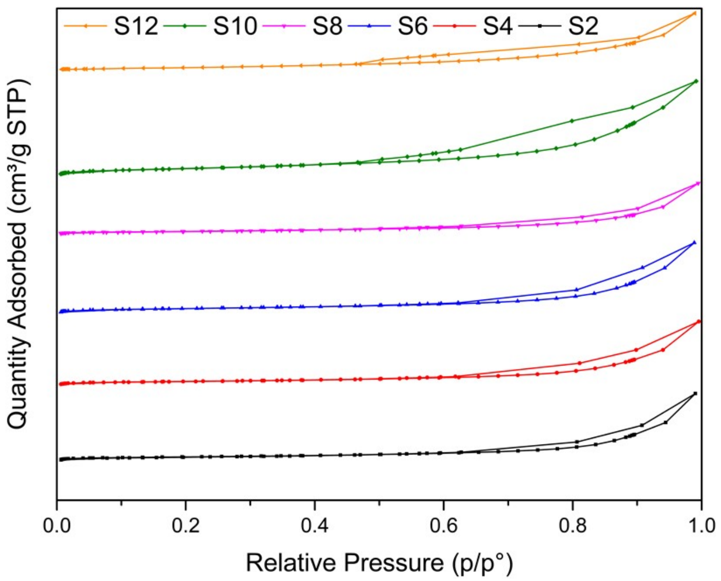

Figure S3. Isotherms for the developed catalysts measured by N_2-sorption analysis.

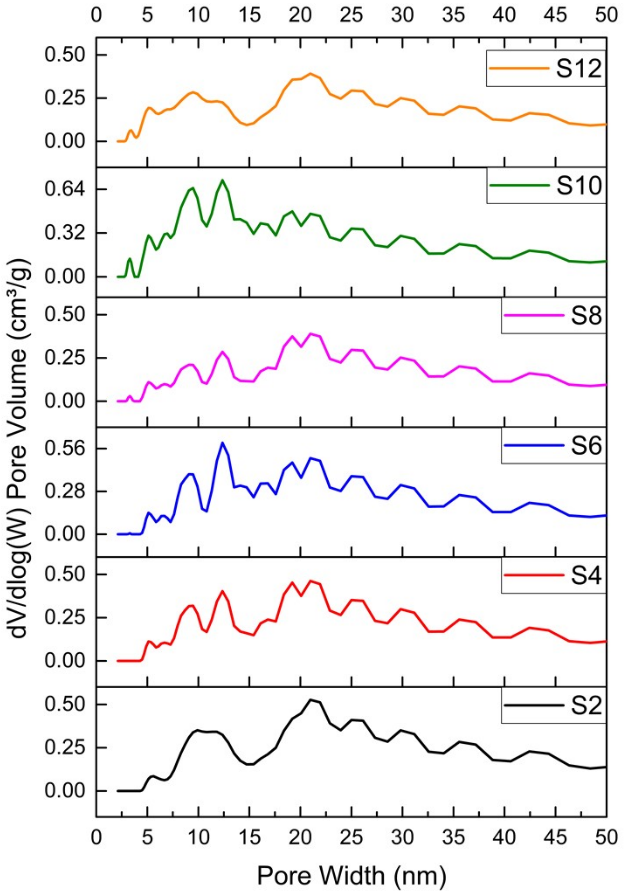

Figure S4. Pore Size Distribution for the developed catalysts measured by N_2-sorption analysis.

Table S1. BET and Pore Size analysis for undoped samples.

Sample	***S_{BET} [m²/g]***	***Micropore area [m²/g]***	***V_t [cm³/g]***	***Pore Size [nm]***	***Si/Al ratio***
**S01: Raw HNT**	65	7	0.276	14.7	1.02
**S03: HNO_3-HNT**	40.5	8	0.23	14.1	1.26
**S05: H_2SO_4-HNT**	62.5	9	0.354	14	1.45
**S07: Alkaline-HNT NaNO3 ball milled**	19	6	0.105	19.5	0.99
**S09: Alkaline-HNT NaOH**	50.19	5.55	0.3208	19.25	0.99
**S11: Alkaline-HNT NaNO3 grinded**	47	7	0.277	13.8	1.04

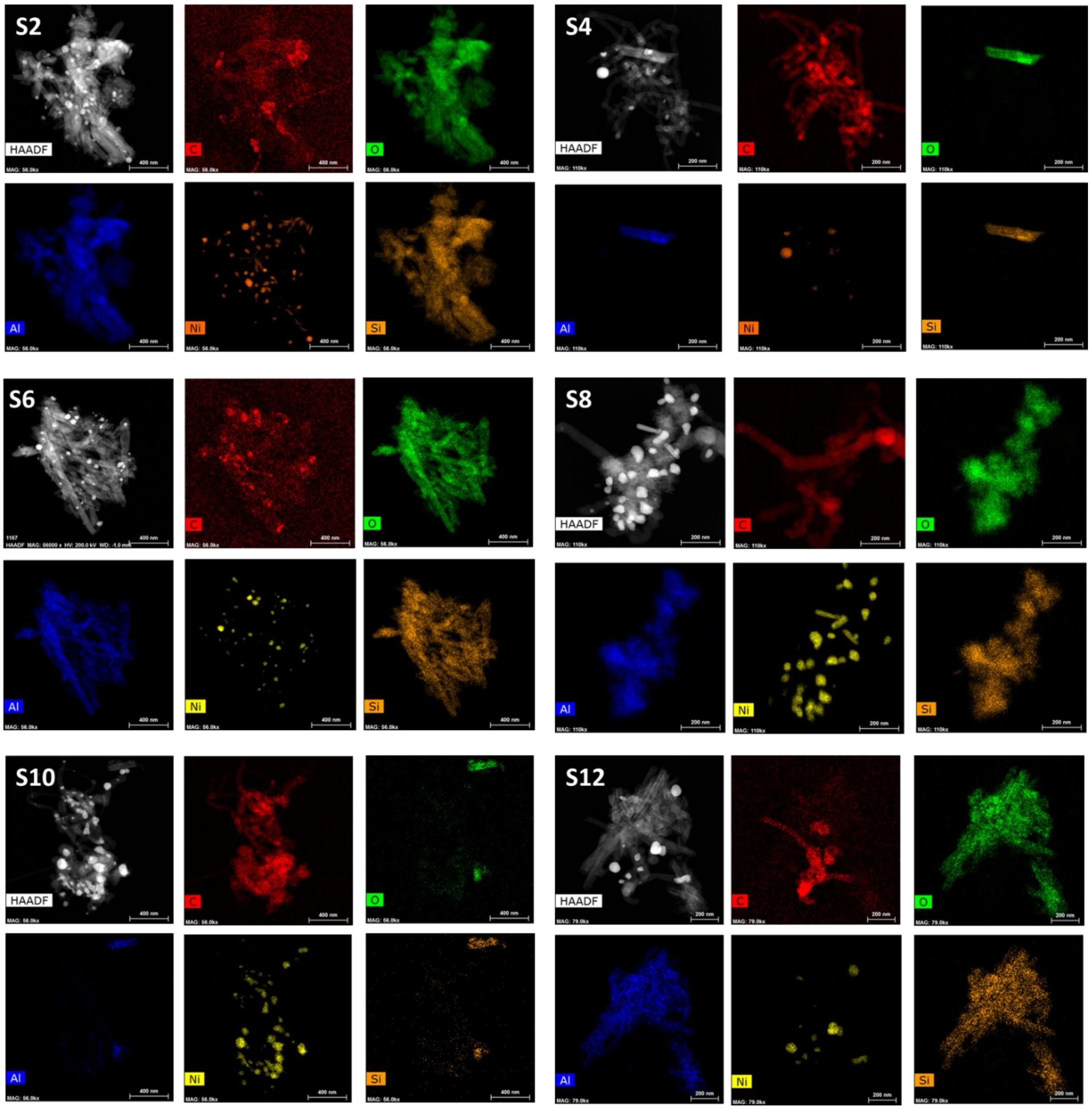

Figure S5. TEM-EDS elemental mapping of Si, Al, O, C and Ni on spent catalysts S2- Ni-Raw HNT, S4- Ni-AHNT (HNO_3), S6-Ni-AHNT (H_2SO_4), S8-Ni-AlHNT (ball milled), S10-Ni-AlHNT (NaOH), and S12-Ni-AlHNT (grinded).

Table S2. Particle size analysis for both fresh and spent catalyst, calculated from TEM images.

Sample	Mean Particle Size [nm]	Standard Deviation	Average Ni particle size increase due to sintering [nm]
**Fresh S2**	7.05	6.64	+42
**Spent S2**	49.22	25.07	
**Fresh S4**	7.35	9.23	+22
**Spent S4**	29.57	19.69	
**Fresh S6**	9.24	12.71	+33
**Spent S6**	42.58	21.77	
**Fresh S8**	30.43	24.16	+17
**Spent S8**	47.44	33.63	
**Fresh S10**	20.88	18.04	+29
**Spent S10**	49.97	38.02	
**Fresh S12**	13.67	15.89	+54
**Spent S12**	67.24	37.29	

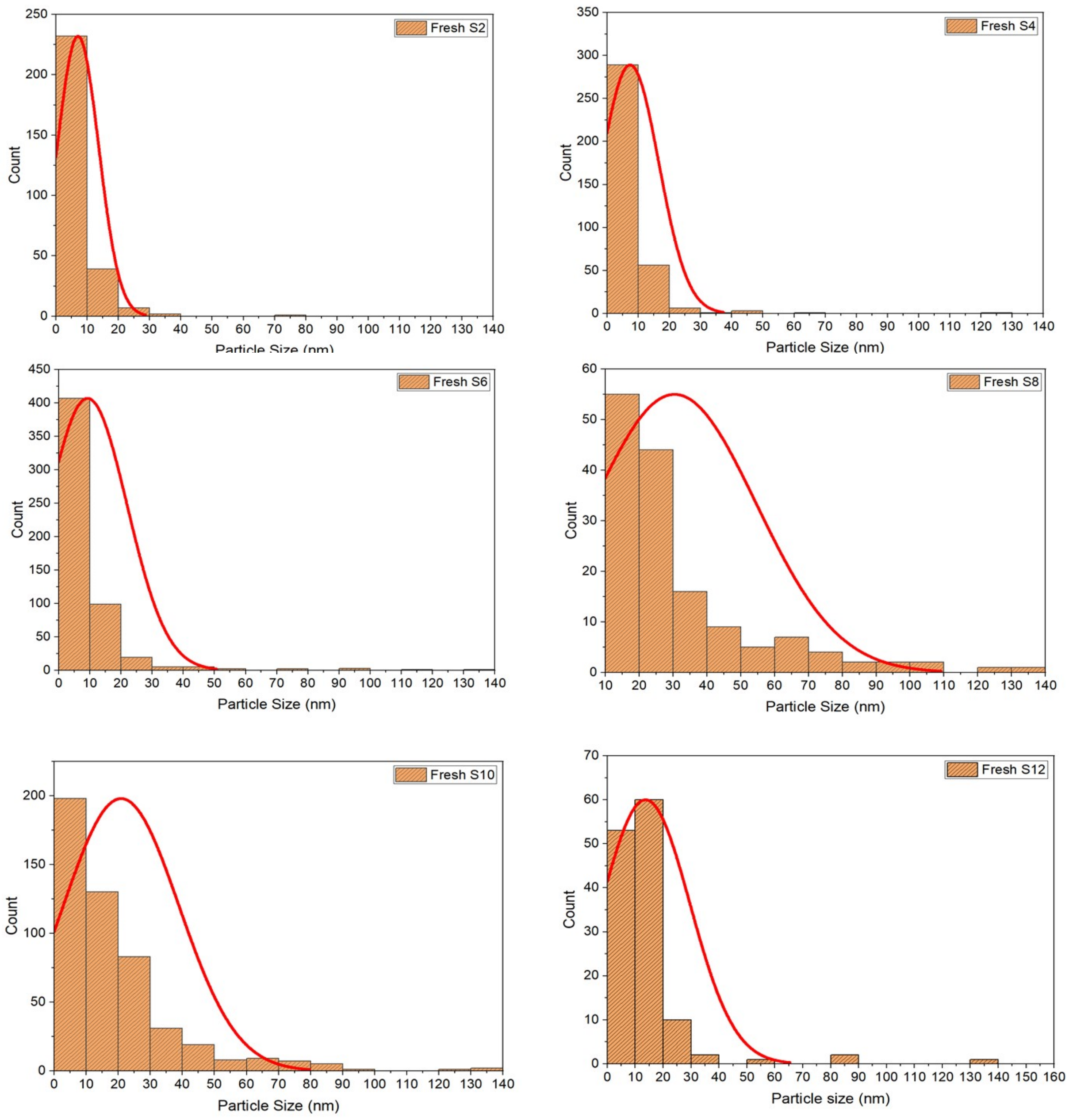

Figure S6. Particle size distribution for the fresh catalysts, calculated from TEM analysis.

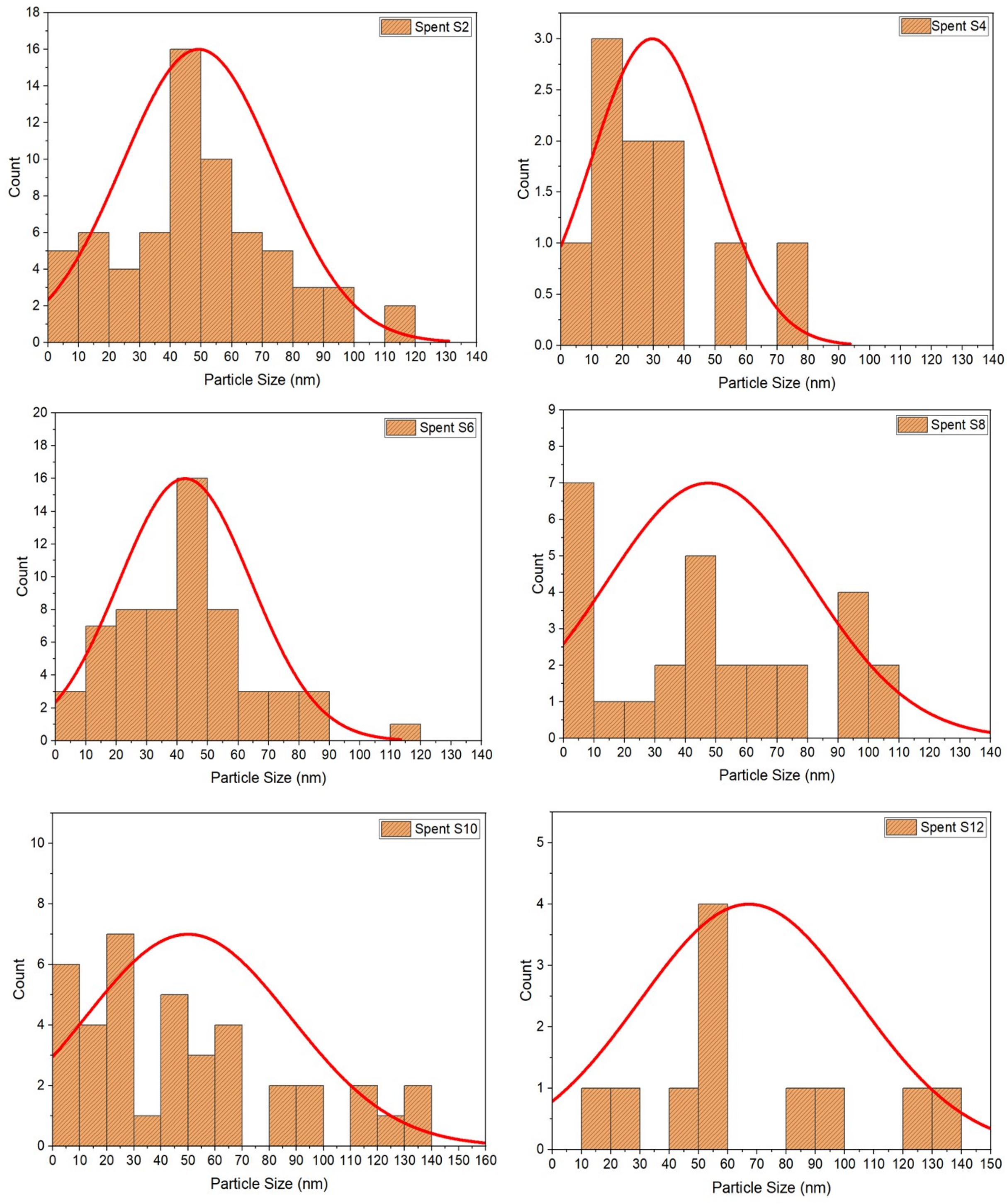

Figure S7. Particle size distribution for the spent catalysts, calculated from TEM analysis.

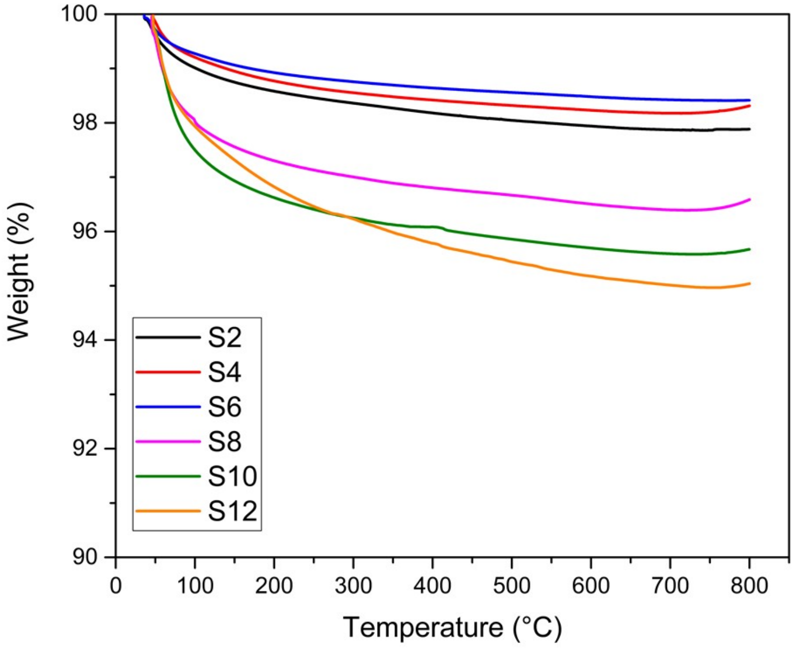

Figure S8. TGA analysis for the reduced catalysts
